# Mobilities of a drop and an encapsulated squirmer

**DOI:** 10.1140/epje/s10189-022-00169-3

**Published:** 2022-02-21

**Authors:** R. Kree, A. Zippelius

**Affiliations:** Georg-August-Universität Göttingen, Institut für Theoretische Physik, Friedrich-Hund-Platz 1, 37077 Göttingen, Germany

## Abstract

**Abstract:**

We have analyzed the dynamics of a spherical, uniaxial squirmer which is located inside a spherical liquid drop at general position $\varvec{r}_s$. The squirmer is subject to an external force and torque in addition to the slip velocity on its surface. We have derived exact analytical expressions for the linear and rotational velocity of the squirmer as well as the linear velocity of the drop for general, non-axisymmetric configurations. The mobilities of both, squirmer and drop, are in general anisotropic, depending on the orientation of $\varvec{r}_s$, relative to squirmer axis, external force or torque. We discuss their dependence on the size of the squirmer, its distance from the center of the drop and the viscosities. Our results provide a framework for the discussion of the trajectories of the composite system of drop and enclosed squirmer.

**Graphical Abstract:**



**Supplementary Information:**

The online version contains supplementary material available at 10.1140/epje/s10189-022-00169-3.

## Introduction

Controlled locomotion on micro- or nanometer scales is of great interest for both, cell biology and microrobotics [1–6]. In the former case, one aims to understand the swimming motion of microorganisms and cell motility. In the latter case, the goals are control and design of microrobots optimized for a variety of biomedical applications. Our focus here is on a composite system, consisting of an active device, encapsulated in a liquid drop.

Such composite systems have been studied experimentally in several different setups, using liquid droplets containing concentrated aqueous solution of bacteria [7–10] in order to understand pattern formation and swimming in a confined geometry. One example are suspensions of *Bacillus subtilis* which form stable vortex patterns inside a liquid drop [7]. In another setup *Escherichia coli* in a water oil emulsion was shown to be able to propel the droplet [8]. Similar propulsion has been observed for bacteria in a liquid droplet, when put into an ordered liquid crystalline state with defects [9]. Yet another example are magnetotactic bacteria which were shown to self-assemble into a rotary motor [10]. In the context of microrobotics, synthetic microswimmers, such as artificial bacterial flagella [11] or photocatalytic particles [12] are able to propel liquid droplets, which is of interest in many biomedical applications, such as targeted drug delivery. The big advantage of self-propulsion is that energy can be supplied by the surroundings; the main disadvantage is lack of control. Therefore, a combination of both, self-propulsion and actuation by external fields, is a promising candidate to achieve optimal control of an otherwise self-propelled composite device.

Theoretical studies of an active particle encapsulated in a drop are based on analytical methods for passive systems, in particular exact solutions in bispherical coordinates [13, 14], the singularity method [15, 16] or multipole expansions [17]. A passive particle encapsulated in a droplet, experiencing external forcing or shear flow has been studied in [18]. Analytical studies of active composite systems have focussed on simple internal active devices. The simplest ones are point forces [19, 20], which can be combined to model pullers and pushers. Alternatively the active device has been taken as a squirmer [21, 22] whose slip velocity generates a flow inside the droplet and thereby can propel it [23, 24]. Marangoni flow on the droplet’s surface provides another driving mechanism, leading to stable co-moving states [25]. The more complex system with many squirmers inside a droplet was studied numerically in [26]; propulsion of the droplet was observed only, when the encapsulated squirmers induced bulk flow in the interior of the droplet.

In a previous paper [27], henceforth denoted by ref. I, we presented an analytical solution for a single-mode squirmer, encapsulated in a drop and displaced from the center of the drop by ${\varvec{a}}$. We only discussed the axisymmetric case, such that both, the symmetry axis of the squirmer and an applied external force, are parallel to the displacement ${\varvec{a}}$. We identified stable, co-moving states of squirmer and drop which can be achieved by an appropriate adjustment of the external force such that squirmer and drop move with the same velocity. These states allow for a controlled manipulation of the viscous drop by external forcing.

Here we extend our analysis and calculate the mobilities of both the squirmer and the drop for general orientations of the displacement $\varvec{a}$ with respect to the symmetry axis of the squirmer and/or the applied external force. For the non-collinear arrangement, the squirmer is subject to a torque with respect to the center of the drop and hence rotates in addition to its linear velocity. We also include an applied external torque, which might be generated by an external magnetic field, provided the active particle is magnetized. In fact propulsion of helical structures by rotating magnetic fields has been discussed in detail [28–30], and biohybrid helical spermbots are interesting candidates for biomedical applications [31]. Electric fields could also provide a torque, if the active particle has a permanent dipole moment.

The linearity of Stokes equation allows us to decompose the analytical calculations into subproblems. We first solve (i) an autonomous swimmer, (ii) a passive particle, which is driven by (iia) an external force or (iib) an external torque. The case of an encapsulated squirmer, subject to an external force and torque, is obtained by superpositions of (i), (iia) and (iib). The analytical solution is constructed in a special geometry, for which the displacement of the squirmer is perpendicular to the squirmer axis or the direction of external force. Then, we superimpose this solution with that of reference I and use frame independence to obtain our results for general displacements and orientations .

The paper is organized as follows: The model is defined in Sect. 2; the analytical method and the solution is presented in Sect. 3. The results of the analytical calculation are the mobilities of the squirmer and the drop as functions of the sizes of particle and drop, the displacement vector and the viscosities. They are presented in Sect. 4 and discussed in Sect. 5.

## Model

**Fig. 1:**
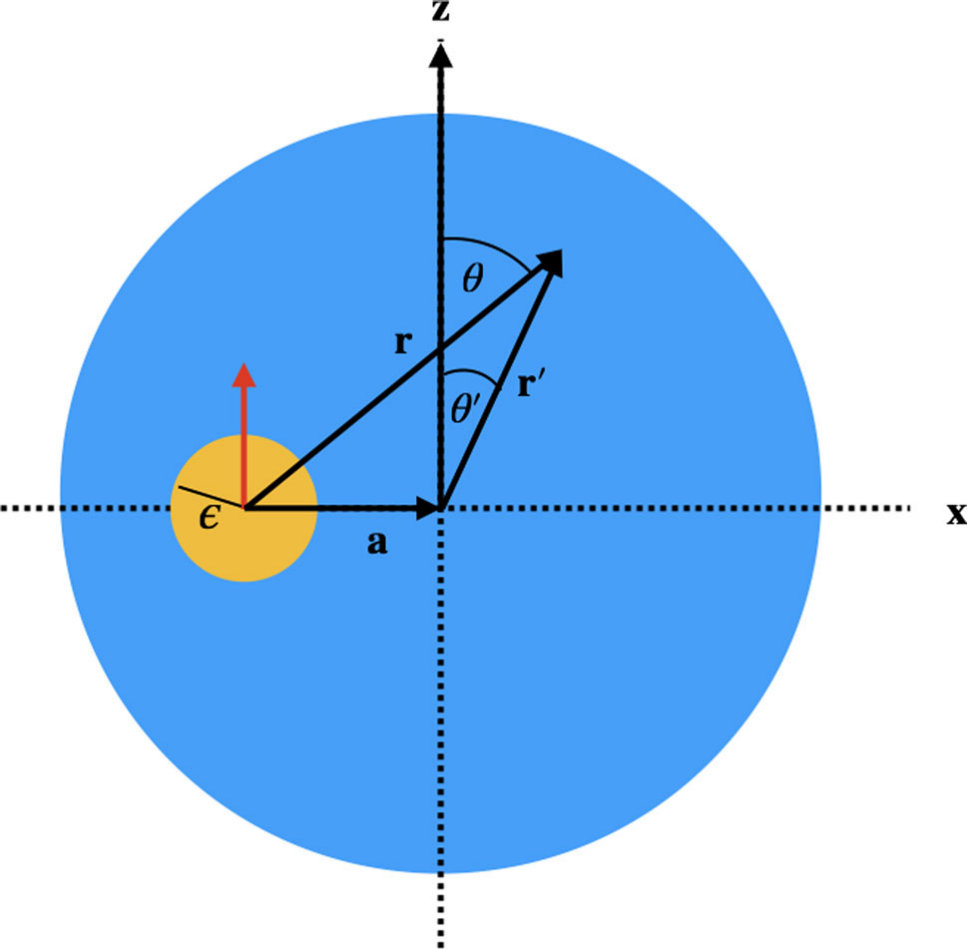
Geometry used in Sect. 2 and 3. Squirmer (yellow) of radius $\epsilon$, encapsulated in a viscous drop (blue) and displaced from the center by $\varvec{a}$. The direction of the displacement is chosen perpendicular to the symmetry axis of the squirmer, $\varvec{n}$, shown as a red arrow and chosen to point along ${\varvec{e}}_z$. If external forces $\varvec{F}_{ext}$ are present, they also point in z-direction and external torques $\varvec{D}_{ext}$ point in y-direction

We study the propulsion of a viscous drop, which is driven by an active device in its interior, as depicted in Fig. 1. The active device is either a squirmer with a tangential slip velocity on its surface (1) or a passive particle, subject to an external force ${\varvec{F}}^{ext}$ and/or torque ${\varvec{D}}^{ext}$ (2), or a combination of both. The active device is modeled as a solid particle of radius $\epsilon$, positioned at ${\varvec{r}}_s=-\varvec{a}$, measured from the center of the drop. We consider *perpendicular* alignment of $\varvec{a}$ and squirmer axis ${\varvec{n}}$ for problem (1) and similarly *perpendicular* alignment of $\varvec{a}$ and ${\varvec{F}}_{ext}$ and $\varvec{D}_{ext}$ for problem (2). We first choose special coordinates with $\varvec{a}=a\varvec{e}_x$, $\varvec{n}=\varvec{e}_z$ and ${\varvec{F}}_{ext}=F_{ext}{\varvec{e}}_z$ and $\varvec{D}_{ext}=D_{ext}\varvec{e}_y$. In all of this and the next section, we will stick to this assignment and postpone a discussion of general relative orientations to Sect. 4. We introduce two frames of reference: one with its origin in the center of the particle (P) and one with its origin in the center of the drop (D). A point has position vector $\varvec{r}$ in the first frame and position vector ${\varvec{r}}^{\prime }={\varvec{r}}-{\varvec{a}}$ in the second (see Fig. 1).

The drop is assumed to be spherical and consists of an incompressible Newtonian fluid with viscosity $\eta ^-$. It is immersed in an ambient Newtonian fluid of viscosity $\eta ^+$ which is at rest in the laboratory frame (LF). The two fluids are assumed to be completely immiscible, and the drop is neutrally buoyant. We choose units of mass, length and time such that the density $\rho _0=1$, the drop radius $R=1$ and the viscosity of the exterior fluid $\eta ^+=1$. We do, however, keep the notation $\eta ^+$, because some results, e.g., the mobility of the drop in the exterior fluid, are more intuitive with the explicit notation. The slip velocity is expanded in spherical harmonics $\chi _{lm}(\varOmega )=P_l^m(\cos {\theta })e^{im\phi }$ in the coordinate system (P) of the squirmer, i.e., the angle $\varOmega =(\theta , \phi )$ has its vertex at the center of the squirmer. The associated Legendre polynomials are denoted by $P_l^m$ and *l* and *m* are integers with $0\le l$ and $-l\le m \le l$. For the purposes of this work, we choose the simplest form of the slip velocity on the surface of the squirmer:

1 $$\begin{aligned} \varvec{v}_{slip}(\varOmega )= -h\sin \theta \varvec{e}_\theta =h\nabla _s\chi _{10}, \end{aligned}$$

where $\nabla _s$ denotes the surface gradient. We only consider the $\ell =1, m=0$ component and choose the axis of the squirmer as the polar axis $\theta =0$. Generalization to $m=\pm 1$ is straightforward as well as the inclusion of chiral flow. In the classical literature [23, 24], Eq. (1) corresponds to a one-mode model. We do not include a second mode (usually referred to as $B_2$, corresponding to $\ell =2$) which would be needed to discriminate between pullers and pushers.

The squirmer generates a flow field inside ($\varvec{v}^-$) and outside ($\varvec{v}^+$) of the drop. For small Reynolds number, the flow fields can be calculated from Stokes equation

2 $$\begin{aligned} \nabla \cdot \varvec{\sigma }^{\pm }=\eta ^{\pm }\nabla ^2\varvec{v}^{\pm }-\nabla p^{\pm }=0 , \end{aligned}$$

and the incompressibility condition $\nabla \cdot \varvec{v}^{\pm }=0$. The viscous stress tensor $\varvec{\sigma }^{\pm }$ is given by its Cartesian components $\sigma _{ij}^{\pm }=-p^{\pm }\delta _{ij}+\eta ^{\pm }(\partial _iv_j^{\pm }+\partial _jv_i^{\pm })$, with the pressure *p* determined from incompressibility.

Stokes equation has to be supplemented by boundary conditions on the surface of the active particle and on the surface of the drop. Given the displacement of the active particle away from the center of the drop, we expect linear as well as rotational motion of the particle (squirmer or dragged passive particle). Hence, the flow field on the surface of the particle in frame (P) takes the general form:

3 $$\begin{aligned} \varvec{v}^-(\varvec{r})=\varvec{v}_{slip}(\varvec{r})+\varvec{U}+\varvec{\omega }\times \varvec{r} \quad \text {for} \quad r=\epsilon , \end{aligned}$$

where $\varvec{U}$ denotes the linear and $\omega$ the angular velocity of the particle. Continuity of the flow field is assumed for points on the surface of the drop in frame (D) with position vector ${\varvec{r}}^{\prime }$

4 $$\begin{aligned} \varvec{v}^-(\varvec{r^{\prime }})=\varvec{v}^+(\varvec{r^{\prime }}) \quad \text {for}\quad r'=1. \end{aligned}$$

The tangential stress is continuous, whereas the normal stress jumps due to a homogeneous surface tension $\gamma _0$, so that

5 $$\begin{aligned} \varvec{e}_{r^{\prime }}\cdot (\varvec{\sigma }_+-\varvec{\sigma }_-)=2\gamma _0\varvec{e}_{r^{\prime }} \quad \text {for} \quad r'=1, \end{aligned}$$

with $\varvec{e}_{r'}=\varvec{r'}/r'$. Once the flow fields $\varvec{v}^{\pm }$ have been determined, the linear velocity of the drop can be computed as an integral over the drop’s surface from

6 $$\begin{aligned} \varvec{v}_{cm}=\frac{3}{4\pi }\int _{r'=1} d^2x \;(\varvec{e}_{r^{\prime }}\cdot \varvec{v}^{\pm })\,\varvec{e}_{r^{\prime }}. \end{aligned}$$

## Analytical solution

Our strategy for the analytical solution is analogous to the one previously used in I. We briefly recall it for consistency. In a first step, the internal flow, $\varvec{v}^-$, is expanded in a complete set of solutions of the Stokes equations in frame (P), and is matched to the slip velocity on the squirmer’s surface. This solution is similar to the flow field of a squirmer in unbounded space, but contains—in addition to fields which are regular at infinity—also those which are regular at the squirmer’s center and would be forbidden in unbounded space.

The boundary conditions on the drop’s surface are easily formulated in the frame (D). Therefore, we seek to expand the flow field $\varvec{v}^-(\varvec{r})$ $=\varvec{v}^-(\varvec{r'} + \varvec{a})$ around the drop’s center in the same set of solutions as used in (P). In contrast to reference I, we consider displacements of the squirmer, which are perpendicular to the squirmer axis or the external force or torque.

### Solutions of Stokes equation

The general solution of Stokes equations in spherical coordinates has first been given in [32] in terms of scalar spherical harmonics. Later [33, 34] they have also been solved using vector spherical harmonics (VSH); a function set, which exists in many variants [35–38]. For our purposes, we use

7 $$\begin{aligned} \varvec{\varPsi }^1_{lm}= & {} \nabla _s\chi _{lm}+l\chi _{lm} \varvec{e}_r \end{aligned}$$

8 $$\begin{aligned} \varvec{\varPsi }^3_{lm}= & {} \nabla _s\chi _{lm}-(l+1)\chi _{lm} \varvec{e}_r \end{aligned}$$

9 $$\begin{aligned} \varvec{\varPsi }^2_{lm}= & {} \varvec{e}_r\times \nabla _s\chi _{lm}, \end{aligned}$$

because they diagonalize the surface Laplacian and form a ($L^2$) complete orthogonal set of functions on the surface of a sphere. Solutions of the Stokes equations can be classified according to whether they are regular at the origin (inner solutions) or regular at infinity (outer solutions). From the VSH functions, we construct a complete set of inner solutions of Stokes equations, which is given by

$$\begin{aligned} \varvec{u}_{lm}^{1<}= & {} \frac{r^{l-1}}{(l+m)!} \varvec{\varPsi }^1_{lm}\\ \varvec{u}_{lm}^{2<}= & {} \frac{r^{l}}{(l+m)!(l+1)} \varvec{\varPsi }^2_{lm}\\ \varvec{u}_{lm}^{3<}= & {} \frac{r^{l+1}}{(l+m)!(2l+1)} \left( \varvec{\varPsi }^1_{lm}+\frac{2l}{(l+1)(2l+3)} \varvec{\varPsi }^3_{lm}\right) , \end{aligned}$$

and outer solutions, which take on the form:

$$\begin{aligned} \mathbf {u}_{lm}^{1>}= & {} \frac{(l-m)!}{r^{l+2}} \varvec{\varPsi }^3_{lm}\\ \varvec{u}_{lm}^{2>}= & {} -\frac{(l-m)!}{lr^{l+1}} \varvec{\varPsi }^2_{lm}\\ \varvec{u}_{lm}^{3>}= & {} \frac{(l-m)!}{r^l(2l+1)} \left( -\varvec{\varPsi }^3_{lm} +\frac{2(l+1)}{l(2l-1)} \varvec{\varPsi }^1_{lm}\right) . \end{aligned}$$

Prefactors have been chosen to simplify expressions in subsequent calculations.

The slip velocity Eq. (1) takes on the form $\varvec{v}_{slip}= h \nabla _s\chi _{10}= (h/3) (\varvec{\varPsi }^3_{10} + 2\varvec{\varPsi }^1_{10})$ in VSH. For displacements $\varvec{a}=a\varvec{e}_x$, we expect to find a solution with $\varvec{U}=U\varvec{e}_z$ and $\varvec{\omega }=\omega \varvec{e}_y$. These velocities are expanded in VSH as follows:

10 $$\begin{aligned} \varvec{U}&=U \varvec{\varPsi }^1_{10} \end{aligned}$$

11 $$\begin{aligned} \varvec{\omega }\times \varvec{r}&=-\frac{i}{2}\omega r (\varvec{\varPsi }^2_{11}+ 2\varvec{\varPsi }^2_{1-1})=r\omega \Im \varvec{\varPsi }^2_{11}, \end{aligned}$$

where $\Im$ denotes the imaginary part. To construct a solution of the boundary value problem, we start from an ansatz with three vector spherical harmonics: $\varvec{\varPsi }^1:=\varvec{\varPsi }^1_{10}$, $\varvec{\varPsi }^3:=\varvec{\varPsi }^3_{10}$ and $\varvec{\varPsi }^2:=\Im \varvec{\varPsi }^2_{11}=(\varvec{\varPsi }^2_{11}+2\varvec{\varPsi }^2_{11-})/(2i)$. The inner and outer solutions take on the form:

12 $$\begin{aligned} \varvec{u}^{1<}:= & {} \varvec{u}^{1<}_{10}=\varvec{\varPsi }^1 \end{aligned}$$

13 $$\begin{aligned} \varvec{u}^{2<}:= & {} (\varvec{u}^{2<}_{11}+2\varvec{u}^{2<}_{1-1})/(2i)=\frac{r}{4}\varvec{\varPsi }^2 \end{aligned}$$

14 $$\begin{aligned} \varvec{u}^{3<}:= & {} \varvec{u}^{3<}_{10}=\frac{r^2}{3} (\varvec{\varPsi }^1+\frac{1}{5} \varvec{\varPsi }^3) \end{aligned}$$

15 $$\begin{aligned} \varvec{u}^{1>}:= & {} \varvec{u}^{1>}_{10}=\frac{1}{r^3} \varvec{\varPsi }^3 \end{aligned}$$

16 $$\begin{aligned} \varvec{u}^{2>}:= & {} (\varvec{u}^{2>}_{11}+2\varvec{u}^{2>}_{1-1})/(2i)=-\frac{1}{r^{2}} \varvec{\varPsi }^2 \end{aligned}$$

17 $$\begin{aligned} \varvec{u}^{3>}:= & {} \varvec{u}^{3>}_{10}=\frac{1}{3r} (-\varvec{\varPsi }^3 +4\varvec{\varPsi }^1) . \end{aligned}$$

The only solutions, which are accompanied by pressure are $\varvec{u}^{3>}$ and $\varvec{u}^{3<}$, and the corresponding pressures are explicitly given by

18 $$\begin{aligned} p^<= 2\eta ^-r\cos {\theta } \qquad \text {and} \qquad p^>= 2\eta ^-\cos {\theta }/r^2,\nonumber \\ \end{aligned}$$

apart from a constant reference pressure.

The general solution of Stokes equation in frame (P) in the interior of the drop is given by a superposition of both, the inner and outer solutions:

19 $$\begin{aligned} \varvec{v}^-= & {} a_1\varvec{u}^{1<}+a_2 \varvec{u}^{2<}+a_3\varvec{u}^{3<}+b_1\varvec{u}^{1>} \nonumber \\&+b_2 \varvec{u}^{2>}+b_3\varvec{u}^{3>}. \end{aligned}$$

The flow field in frame (D) outside of the drop is given by

20 $$\begin{aligned} \varvec{v}^+=c_1\varvec{u}^{1>}++c_2\varvec{u}^{2>}+ c_3\varvec{u}^{3>}. \end{aligned}$$

This Ansatz involves nine parameters, which have to be determined by the boundary conditions.

### Drop velocity

To express the drop velocity $\varvec{v}_{cm}$ by the parameters of the solution, we insert Eq. (20) into Eq. (6). With $\varvec{e}_{r'}\cdot \varvec{\varPsi ^1}=\cos \theta$ and $\varvec{e}_{r'}\cdot \varvec{\varPsi }^3=-2\cos \theta$, we find

21 $$\begin{aligned} \varvec{v}_{cm}=v_{cm}\varvec{e}_z=2(c_3-c_1)\varvec{e}_z. \end{aligned}$$

### Boundary condition on the surface of the squirmer

Given the interior flow field in the form of an expansion around the squirmer’s center (19) in frame (P), we can easily fulfill the boundary condition on the squirmer’s surface. Plugging our Ansatz into Eq. (3), we obtain three equations:

22 $$\begin{aligned} a_1+\frac{\varepsilon ^2}{3}a_3+\frac{4}{3\varepsilon }b_3=\,&U+\frac{2}{3}h \end{aligned}$$

23 $$\begin{aligned} \frac{\varepsilon ^2}{15}a_3+\frac{1}{\varepsilon ^3}b_1-\frac{1}{3\varepsilon }b_3=\,&\frac{1}{3}h. \end{aligned}$$

24 $$\begin{aligned} \frac{\varepsilon }{4}a_2-\frac{b_2}{\varepsilon ^2}=\,&\varepsilon \omega , \end{aligned}$$

relating the coefficients of the interior flow field to the activity of the squirmer. Further six equations are provided by the boundary conditions on the drop’s surface Eqs. (4, 5). However, before we can use them, we have to shift the internal flow $\varvec{v}^-$, given in frame (P) to its representation in frame (D).

### Translations

To express the vector field $\varvec{v}^-$, given in terms of the solutions Eqs. (12–17) in frame (P), on the surface of the drop in frame (D), we derive a generalization of the corresponding transformations for the solid scalar spherical harmonics, which can be found in in [39]. These translations are easily worked out by hand for the $\ell =1$ components of the flow, as is explained in Appendix A, which also contains an example. Here we just state the results:

$$\begin{aligned} \varvec{u}^{1<}(\varvec{r'}+a\varvec{e}_x)= & {} \varvec{u}^{1<}(\varvec{r'})\\ \varvec{u}^{2<}(\varvec{r'}+a\varvec{e}_x)= & {} \varvec{u}^{2<}(\varvec{r'}) - \frac{a}{4}\varvec{u}^{1<}(\varvec{r'})\\ \varvec{u}^{3<}(\varvec{r'}+a\varvec{e}_x)= & {} \varvec{u}^{3<}(\varvec{r'})+ \frac{2a^2}{5}\varvec{u}^{1<}(\varvec{r'})\\&-2a \varvec{u}^{2<}(\varvec{r'}) +...\\ \varvec{u}^{1>}(\varvec{r'}+a\varvec{e}_x)= & {} \varvec{u}^{1>}(\varvec{r'}) +...\\ \varvec{u}^{2>}(\varvec{r'}+a\varvec{e}_x)= & {} \varvec{u}^{2>}(\varvec{r'}) - \frac{a}{2}\varvec{u}^{1>}(\varvec{r'})+... \\ \varvec{u}^{3>}(\varvec{r'}+a\varvec{e}_x)= & {} \varvec{u}^{3>}(\varvec{r'})+ \frac{2a^2}{5}\varvec{u}^{1>}(\varvec{r'})\\&-a \varvec{u}^{2>}(\varvec{r'}) +... \end{aligned}$$

The ellipsis (...) denote non-vanishing terms with $l\ge 2$. These terms do not contribute to the velocities of squirmer and drop, but will in general lead to deformations of the drop’s spherical shape. In the present work, we do not study this part of the flow.

### Boundary conditions on the surface of the drop

Given the translated velocity fields, we can evaluate the internal flow $\varvec{v}^-(\varvec{e}_{r^{\prime }}+a\varvec{e}_x)$ at the boundary of the drop ($r'=1$), as needed for the second boundary condition Eq. (4). Continuity of the velocity across the drop’s surface implies

25 $$\begin{aligned}&a_1+\frac{a_3}{3}\left( 1+\frac{6}{5} a^2\right) +\frac{4}{3}(b_3-c_3)-\frac{a}{4}a_2=0 \end{aligned}$$

26 $$\begin{aligned}&b_1-\frac{b_3}{3}\left( 1-\frac{6}{5} a^2\right) +\frac{a_3}{15}-\frac{a}{2}b_2-c_1+\frac{c_3}{3}=0 \end{aligned}$$

27 $$\begin{aligned}&-\frac{a}{2}a_3+\frac{a_2}{4}+ab_3-b_2+c_2=0. \end{aligned}$$

To fulfill the balance of forces on the drop’s surface Eq. (5), we need to compute the tractions $\varvec{t}=-p\varvec{e}_r + \varvec{t}_{vis}$. The viscous part is obtained for any Stokes flow $\varvec{u}^\pm$, using the identity

28 $$\begin{aligned} \varvec{t}_{vis}^{\pm }=\eta ^{\pm }\left( 2 \frac{\partial }{\partial r}+{\varvec{e}}_r\times \nabla \times \right) {\varvec{u}}^{\pm }. \end{aligned}$$

Together with the pressure contribution, we find for the tractions in the interior of the fluid

29 $$\begin{aligned} \varvec{t}^{1<}= & {} 0 \end{aligned}$$

30 $$\begin{aligned} \varvec{t}^{2<}= & {} 0 \end{aligned}$$

31 $$\begin{aligned} \varvec{t}^{3<}= & {} \frac{3\eta ^-r}{5}\varvec{\varPsi }^3 \end{aligned}$$

32 $$\begin{aligned} \varvec{t}^{1>}= & {} -\frac{6\eta ^-}{r^4}\varvec{\varPsi }^3 \end{aligned}$$

33 $$\begin{aligned} \varvec{t}^{2>}= & {} \frac{3\eta ^-}{r^3}\varvec{\varPsi }^2 \end{aligned}$$

34 $$\begin{aligned} \varvec{t}^{3>}= & {} \frac{2\eta ^-}{r^2}(\varvec{\varPsi }^3-\varvec{\varPsi }^1), \end{aligned}$$

represented in the frame (P). Here, the superscript $<(>)$ refers to the flow fields $\varvec{u}^< (\varvec{u}^>)$ and the pressure $p^< (p^>)$. Since the tractions are linear functions of the velocities, the transformation to the frame (D) is easily obtained from the transformation of the velocities:

35 $$\begin{aligned} \varvec{t}^{2>}= & {} =\frac{3\eta ^-}{r^3}\varvec{\varPsi }^2+\frac{3a\eta ^-}{r^4}\varvec{\varPsi }^3 \end{aligned}$$

36 $$\begin{aligned} \varvec{t}^{3>}= & {} \frac{2\eta ^-}{r^2}(\varvec{\varPsi }^3-\varvec{\varPsi }^1)-\frac{12a^2\eta ^-}{5r^4}\varvec{\varPsi }^3 -\frac{3a\eta ^-}{r^3 }\varvec{\varPsi }^2\nonumber \\ \end{aligned}$$

All other tractions turn out to be unaffected by the translation.

The above tractions are plugged into the third boundary condition Eq. (5), implying 3 more linear equations for the yet unknown coefficients

37 $$\begin{aligned}&b_3=\frac{c_3}{\lambda } \end{aligned}$$

38 $$\begin{aligned}&\frac{3}{5}a_3-6b_1+3ab_2+2b_3(1-\frac{6}{5}a^2)=\frac{2(c_3-3c_1)}{\lambda }\nonumber \\ \end{aligned}$$

39 $$\begin{aligned}&b_2-ab_3=\frac{c_2}{\lambda }, \end{aligned}$$

where $\lambda =\eta ^-/\eta ^+$ denotes the viscosity contrast.

### Force and torque balance

The boundary conditions on the surface of the squirmer and the drop provide nine linear equations. Force and torque balance yield two more equations, so that all unknowns, the nine coefficients of the general solution and *U* and $\omega$, are uniquely determined.

An external force acting on the particle has to be balanced by the total viscous force: $\varvec{F}_{visc}+\varvec{F}_{ext}=\varvec{0}$. The total viscous force $\varvec{F}_{visc}$ can be expressed as an integral of the tractions over the surface of a large sphere of radius $R\gg 1$)

40 $$\begin{aligned} \varvec{F}_{visc}=\lim _{R\rightarrow \infty } \int _{R} d^2 x \;\varvec{t}^+. \end{aligned}$$

The only flow term contributing to this expression is $c_3\varvec{u}^{3>}$ which is $\sim 1/r$, so that

41 $$\begin{aligned} \varvec{F}_{visc}= -8\pi \eta ^+ c_3\varvec{e}_z=-\varvec{F}_{ext}. \end{aligned}$$

Hence, force balance determines the coefficient $c_3$.

In the balance of torque $\varvec{D}_{visc} + \varvec{D}=\varvec{0}$, the viscous part is determined from

42 $$\begin{aligned} \varvec{D}_{visc}=\lim _{R\rightarrow \infty } \int _R d^2 x \;\varvec{r}\times \varvec{t}^+. \end{aligned}$$

Note that this torque is calculated in frame (D). The only flow term contributing to this expression is $c_2\varvec{u}^{2>}$ which falls off as $\sim 1/r^2$, so that in our geometry $\varvec{D}_{visc}=D_{visc}\varvec{e}_y$ with

43 $$\begin{aligned} D_{visc}=8\pi \eta ^+c_2\varvec{e}_y. \end{aligned}$$

The exerted torque in this frame is given by $\varvec{D}=\varvec{D}_F + \varvec{D}_{ext}$. The first term $\varvec{D}_F= -\varvec{a}\times \varvec{F}_{ext}$ arises from any moment-free force distribution with total force $\varvec{F}_{ext}$. In our special geometry, the torque balance becomes

44 $$\begin{aligned} 8\pi \eta ^+c_2 + aF_{ext} + D_{ext}=0, \end{aligned}$$

which fixes the parameter $c_2$.

## Mobilities of squirmer and drop

The analytical solution of the linear system of Eqs. (22–24), (25–27) and (37–39) is discussed here for three different situations:

- an autonomous squirmer without applied external force or torque
- a passive particle (no slip velocity) dragged by an external force
- a passive particle (no slip velocity) subject to an external torque.

We extract the analytical expressions for the mobilities, relating $\varvec{F}_{ext}, \varvec{D}_{ext}$ and the squirmer activity *h* to the velocities $\varvec{U},\varvec{\omega }$ of the particle and $\varvec{v}_{cm}$ of the drop. Combining these results with reference I and using frame independence, we then obtain the mobility tensors of the particle and the drop for each of the three cases. The complete analytical expressions for all the mobility tensors, including those from reference I can be worked out by hand (and have been checked by symbolic computing using *SymPy* [40]). They are summarized in Appendix B. More general situations, representing a squirmer subject to both, external force and torque, which drives its enclosing drop, can be obtained by linear superposition.

### Encapsulated squirmer

The activity of the squirmer is conveniently characterized by its velocity $U_0=-2h/3$ in an unbounded fluid. For the autonomous swimmer, force and torque balance imply $b_3=c_3=b_2=c_2=0$. The remaining linear equations are easily solved and yield

45 $$\begin{aligned}&U =\,\zeta ^{\perp }_h(a^2, \epsilon , \lambda )\, U_0 \end{aligned}$$

46 $$\begin{aligned}&\zeta ^{\perp }_h(a^2, \epsilon , \lambda ) = -\frac{3}{2N}(\lambda -1)\epsilon ^3 a^2 + \zeta ^{\perp }_h(0, \epsilon , \lambda ). \end{aligned}$$

with

47 $$\begin{aligned} N=\,&2\epsilon ^5(\lambda -1) +3\lambda +2, \end{aligned}$$

48 $$\begin{aligned} N\zeta ^{\perp }_h(0,\epsilon , \lambda ) =\,&3\lambda +2 - (\lambda - 1)\epsilon ^3 (3 \epsilon ^2 -5) \end{aligned}$$

The offset of the squirmer from the center of the drop in a direction perpendicular to its symmetry axis (see Fig. 1) gives rise to an angular velocity $\varvec{\omega }=\omega \varvec{e}_y$ of the particle with

49 $$\begin{aligned} \omega =-\kappa _h(\epsilon , \lambda )a U_0=-\frac{15\epsilon ^3 (\lambda -1)}{2N}aU_0 . \end{aligned}$$

The angular velocity vanishes linearly with *a*.

The drop moves in the direction of the symmetry axis of the squirmer, $\varvec{v}_{cm}=v_{cm}\varvec{e}_z$, obtained from Eq. (21), with

50 $$\begin{aligned} v_{cm}= \mu ^{\perp }_hU_0=\frac{5\epsilon ^3\lambda }{N}U_0. \end{aligned}$$

In I we considered an autonomous swimmer, which is displaced from the center *parallel* to its symmetry axis $\mathbf {e}_z$. We now combine these results with our new ones for perpendicular alignment to obtain the mobilities for general orientations of displacement $\varvec{a}$ and symmetry axis of the squirmer $\varvec{n}=\varvec{U}_0/U_0$, and we write the linear superposition of both results in coordinate free form as follows:

51 $$\begin{aligned} \varvec{U}&= \hat{\varvec{\zeta }}_h \varvec{U}_0 \end{aligned}$$

52 $$\begin{aligned} \varvec{v}_{cm}&= \hat{\varvec{\mu }}_h \varvec{U}_0 \end{aligned}$$

53 $$\begin{aligned} \varvec{\omega }&=\kappa _h \varvec{a}\times \varvec{U}_0. \end{aligned}$$

The resulting mobility tensor $\hat{\varvec{\zeta }}_h$ is symmetric and uniaxial with respect to the $\varvec{a}$-direction $\varvec{e}_{||}=\varvec{a}/a$, i.e.,

54 $$\begin{aligned} \hat{\varvec{\zeta }}_h= \zeta ^{||}_h(a^2,\epsilon ,\lambda ) \varvec{e}_{||}\otimes \varvec{e}_{||} + \zeta ^{\perp }_h(a^2, \epsilon , \lambda )(\hat{\varvec{1}}- \varvec{e}_{||}\otimes \varvec{e}_{||}).\nonumber \\ \end{aligned}$$

The longitudinal component $\zeta ^{||}_h$ follows from Eqs. (38, 39) of I and is listed in Appendix B. The anisotropy vanishes trivially for $a=0$, when the encapsulated particle is located at the center of the drop [21].

The mobility of the drop turns out to be isotropic, i.e., $\mu ^{\perp }_{h}=\mu ^{||}_{h}=\mu _h$ and $\hat{\varvec{\mu }}_h=\mu _h\varvec{1}$. Rotation of the drop is only observed for a displacement $\varvec{a}$ with a nonzero component perpendicular to the symmetry axis $\varvec{n}$ of the squirmer. The corresponding mobility tensor is uniaxial but anti-symmetric, so that it is determined by a single coefficient $\kappa _h$, which can be read off from Eq. (49).

### Passive particle dragged by an external force

Next, we consider a passive particle ($h=0$), which is dragged by an external force $\varvec{F}_{ext}=F_{ext}\varvec{e}_z$, perpendicular to its displacement $\varvec{a}=a\varvec{e}_x$ from the center of the drop. The coefficients $c_2,c_3$ are determined by the external force. Solving for the remaining coefficients, we find

55 $$\begin{aligned} \varvec{v}_{cm}=\,&v_{cm}\varvec{e}_z =\,\mu _F^{\perp }\varvec{F}_{ext} \end{aligned}$$

56 $$\begin{aligned} \varvec{U}=\,&U\varvec{e}_z =\,\zeta _F^{\perp }\varvec{F}_{ext} \end{aligned}$$

57 $$\begin{aligned} \varvec{\omega }=\,&\omega \varvec{e}_y =\,\kappa _F\varvec{a}\times \varvec{F}_{ext} \end{aligned}$$

The coefficients $\mu _F^{\perp },\zeta _F^{\perp },\kappa _F$ are given in Eqs. (80, 89, 93) of Appendix B. A finite angular velocity of the particle is due to the torque (in frame D) exerted by the external force due to a finite displacement of the particle from the center of the drop.

We proceed as in the previous subsection: we combine the above results for perpendicular alignment of $\varvec{a}$ and $\varvec{F}_{ext}$ with those of ref. I for parallel alignment. General orientations of $\varvec{a}$ and $\varvec{F}_{ext}$ then give rise to mobility tensor relations, which read in coordinate free representation as follows:

58 $$\begin{aligned} \varvec{U}&= \hat{\varvec{\zeta }}_F \varvec{F}_{ext} \end{aligned}$$

59 $$\begin{aligned} \varvec{v}_{cm}&= \hat{\varvec{\mu }}_F \varvec{F}_{ext} \end{aligned}$$

60 $$\begin{aligned} \varvec{\omega }&=\kappa _F \varvec{a}\times \varvec{F}_{ext}. \end{aligned}$$

The tensors $\hat{\varvec{\zeta }}_F$ and $\hat{\varvec{\mu }}_F$ are symmetric and uniaxial with respect to the displacement $\varvec{a}$. The longitudinal components follow from Eqs. (40, 41) of I and are recalled in Appendix B.

### Passive particle subject to an external torque

Finally, we consider a passive particle ($h=0$) with no applied force ($\varvec{F}_{ext}=0$), subject to an external torque $\varvec{D}_{ext}$ in frame P. To construct the most general case, we must discuss both perpendicular and parallel alignment of torque and displacement, but the latter case has not yet been included in our discussion. It requires an extension of the calculations of reference I, which is given in Appendix C.

For perpendicular alignment and in agreement with the coordinates chosen in Sect. 3, we choose $\varvec{D}_{ext}=D_{ext}\varvec{e}_y$, so that $\varvec{\omega }=\omega \varvec{e}_y$ and $\varvec{U}=U\varvec{e}_z$. In the absence of an applied external force, we have $b_3=c_3=0$. Torque balance determines the coefficient $c_2$ according to Eq. (43) and hence also $b_2=c_2/\lambda$ according to Eq. (39). The transverse mobilities in the equations

61 $$\begin{aligned} U&= \zeta _D^\perp (a^2, \epsilon , \lambda ) D_{ext}a \end{aligned}$$

62 $$\begin{aligned} v_{cm}&= \mu _D^\perp (a^2, \epsilon , \lambda ) D_{ext}a \end{aligned}$$

63 $$\begin{aligned} \omega&=\kappa ^\perp _D(a^2, \epsilon , \lambda ) D_{ext} \end{aligned}$$

are explicitly given in Eqs. (85, 91, 96) of Appendix B.

The configuration with parallel alignment of torque and displacement leads to a spinning motion of the particle around its direction of propulsion. Its calculation requires an extension of the analysis given in reference I, which is presented in Appendix C. The result is $U=0$ and $\omega =\kappa _D^{||}D_{ext}$ with

64 $$\begin{aligned} \kappa _D^{||}= \frac{1}{8\pi \epsilon ^3\eta ^-}\big ( \epsilon ^3(\lambda -1) +1\big ) \end{aligned}$$

In coordinate free representation, the relations between $\varvec{D}_{ext}$ and the velocities take on the form

65 $$\begin{aligned} \varvec{U}&= {\zeta }_D \varvec{a}\times \varvec{D}_{ext} \end{aligned}$$

66 $$\begin{aligned} \varvec{v}_{cm}&= {\mu }_D \varvec{a}\times \varvec{D}_{ext} \end{aligned}$$

67 $$\begin{aligned} \varvec{\omega }&=\hat{\varvec{\kappa }}_D \varvec{D}_{ext}, \end{aligned}$$

with a symmetric uniaxial tensor $\hat{\varvec{\kappa }}_D$.

## Discussion

The general mobility tensors are obtained by superposition of the 3 special cases worked out above and will now be used to discuss the general motion of drop and encapsulated particle.

### Motion of the drop

A linear velocity of the drop is generated by all three driving mechanisms: slip velocity of the squirmer, external force and external torque

68 $$\begin{aligned} \varvec{v}_{cm} =\mu _h\varvec{U}_0+\hat{\varvec{\mu }}_F \varvec{F}_{ext} +\mu _D\varvec{a}\times \varvec{D}_{ext} \end{aligned}$$

The response to active slip, as characterized by $\mu _h$, is completely isotropic and independent of *a*. In other words, the linear velocity of the drop with an encapsulated squirmer only depends on the size $\epsilon$ of the squirmer and the viscosity contrast $\lambda$. For small $\epsilon$, it vanishes proportional to the volume of the squirmer.

If the drop is driven by an external force, acting on a passive encapsulated particle, then the response is anisotropic and characterized by the uniaxial tensor: $\hat{\varvec{\mu }}_F= \mu ^{||}_F(a^2) \varvec{e}_{||}\otimes \varvec{e}_{||} + \mu ^{\perp }_F(a^2)(\varvec{1}- \varvec{e}_{||}\otimes \varvec{e}_{||})$. The ratio of the two mobilities is given by

$$\begin{aligned}&\frac{\mu ^{||}_F}{\mu ^{\perp }_F}=\frac{G-3a^2}{G-6a^2}\quad \text {with}\\&G=4\epsilon ^4(\lambda -1)-5\epsilon ^2+3(2\lambda +3) \end{aligned}$$

so that the velocity of the drop is always larger for parallel alignment of force and displacement. The mobility of the drop remains finite as the size of the particle goes to zero and in fact coincides with the mobilities derived previously [19] for a point force inside a drop. Finally, a torque exerted on the encapsulated particle, also propels the drop, provided the particle is placed off center.

**Fig. 2:**
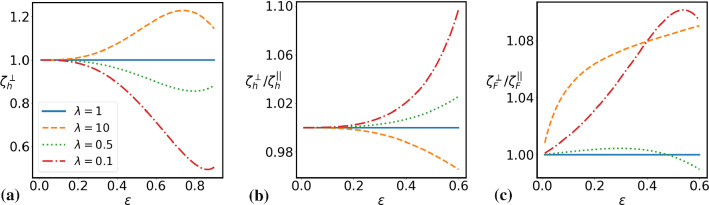
Mobility of a squirmer (**a**) and mobility anisotropy of a squirmer (**b**), and a passive particle dragged by an external force (**c**), *vs.* radius of the particle for different viscosity contrasts $\lambda$, as shown in the legend of (**a**). In (**a**) the distance of the particle from the drop’s center is $r_s=0.1$, in (**b**) and (**c**) it is $r_s=0.4$

### Linear velocity of the particle

The general propulsion velocity of the encapsulated particle is given by

69 $$\begin{aligned} \varvec{U} =\hat{\varvec{\zeta }}_h \varvec{U}_0+\hat{\varvec{\zeta }}_F \varvec{F}_{ext} +{\zeta }_D \varvec{a}\times \varvec{D}_{ext}. \end{aligned}$$

The response of the particle to either an applied force or to a nonzero slip is in general anisotropic. If only an active slip is present or only an external force is applied, the velocity is not in the direction of the squirmer axis or the external force. The reason for this anisotropy is the response flow due to reflection at the interface, which is not concentric around the particle. The anisotropy therefore vanishes as $\epsilon \rightarrow 0$, or equivalently as the radius of the drop goes to infinity. In that limit we recover the result for the squirmer in free space, $\varvec{U}= \varvec{U}_0$, and the result for the mobility of a passive particle dragged by an external force, $\varvec{U} =\frac{1}{6\pi \eta ^{-}\epsilon } \varvec{F}_{ext}$, in leading order in $\epsilon$.

If the squirmer fills the whole drop ($\epsilon \rightarrow 1$), we also find $\varvec{U}\rightarrow \varvec{U}_0$, implying nonmonotonic behavior of $\hat{\varvec{\zeta }}_h$ as a function of $\epsilon$. In Fig. 2a, we show $\zeta _h^{\perp }$ as a function of $\epsilon$ for several values of $\lambda =\eta ^-/\eta ^+$ and $r_s=|\varvec{r}_s|=0.1$.

One clearly observes nonmonotonic behavior for all $\lambda$. Furthermore, if the interior of the drop has a higher viscosity than the outside ($\lambda >1$), the drop moves faster than in free space, because the frictional forces in the interior are larger than in the exterior region. The opposite behavior is observed for $\lambda <1$, i.e., a less viscous interior. In Fig. 2b and c, we illustrate the anisotropy of the response. The ratio $\zeta _h^{\perp }/\zeta _h^{||}$ is shown in Fig. 2b as a function of $\epsilon$ for several values of $\lambda$ and $r_s=0.4$ in comparison with the isotropic case which is realized for $\lambda =1$. Both cases, $\zeta _h^{\perp }\lessgtr \zeta _h^{||}$ are possible, depending on whether $\eta ^+\lessgtr \eta ^-$.

If a passive particle dragged by $\varvec{F}_{ext}$ fills the whole drop ($\epsilon \rightarrow 1$), one obtains $\varvec{U} =\frac{1}{6\pi \eta ^{+}\epsilon } \varvec{F}_{ext}$, as expected. The dependence of the anisotropy on the viscosity contrast $\lambda$ is more subtle, as can be seen in Fig. 2c, where we plot the ratio of perpendicular to parallel mobility for an applied force, $\zeta _F^{\perp }/\zeta _F^{||}$. For $\lambda <1$, the ratio becomes a non-monotonic function of $\epsilon$, and it may show both possibilities $\zeta _h^{\perp }\lessgtr \zeta _h^{||}$, as illustrated by $\lambda =0.5$ in Fig. 2c.

### Rotational velocity of the particle

All 3 driving mechanisms, slip, external force and external torque, give rise to a rotation of the particle:

70 $$\begin{aligned} \varvec{\omega } =\kappa _h\varvec{a}\times \varvec{U}_0+\kappa _F\varvec{a}\times \varvec{F}_{ext}+ \hat{\varvec{\kappa }}_D \varvec{D}_{ext}, \end{aligned}$$

The rotational mobility due to slip vanishes as the volume of the squirmer, i.e., there is no rotational motion of a squirmer in free space. Note that we have not included a chiral component of the slip which has been discussed for a squirmer in unbounded space [41].

An external torque causes a rotation of the particle with in general anisotropic mobilities $\kappa _D^{||}\ne \kappa _D^{\perp }$. In free space, i.e., in the limit $R\rightarrow \infty$, the response becomes isotropic and reduces to the rotational mobility in free space: $\kappa _D^{||}=\kappa _D^{\perp }=\frac{1}{8\pi \eta ^-\epsilon ^3}$.

## Conclusions and outlook

We have analyzed the dynamics of a solid particle, encapsulated in a drop and displaced from the drop’s center by a general vector $\varvec{a}$ (non-axisymmetric configuration). Several driving mechanisms have been considered. Either the solid particle is a (uniaxial) squirmer, driven by an active slip or it is subject to an external force or to an external torque, or any combination thereof. We have derived analytical expressions for the translational and rotational mobilities, i.e., the linear and rotational velocity of the squirmer as well as the linear velocity of the drop as functions of translation vector $\varvec{a}$, particle radius $\epsilon$ and viscosity contrast $\lambda =\eta ^-/\eta ^+$. Our analytical method is adapted to mobility problems in spherical geometries, for which it is simple and straightforward. It can easily be generalized to more complex squirmers, which possess chiral components and/or higher *l*-components of active slip velocity. The obtained results provide a first step towards controlled locomotion of an (active) particle, encapsulated in a spherical liquid drop. Based on the general results for the linear ($\varvec{U}$) and rotational ($\varvec{\omega }$) velocity of the particle as well as the linear velocity of the drop ($\varvec{v}_{cm}$), one has to solve the equations of motion for the particle in the rest frame of the drop:

71 $$\begin{aligned} {\dot{\varvec{r}}_s}&=\varvec{u}=\varvec{U}-\varvec{v}_{cm} \end{aligned}$$

72 $$\begin{aligned} {\dot{\varvec{n}}}&=\varvec{n}\times \varvec{\omega }. \end{aligned}$$

Together with the equation for $\varvec{v}_{cm}$, one thereby obtains the trajectories of drop and squirmer. Adjusting the external force and torque, should allow to steer the composite system to designed places, as required by drug delivery or more generally in the context of microrobotics.

### Supplementary Information

Below is the link to the electronic supplementary material.

Supplementary file 1 (py 8 KB)

## Data Availability

This manuscript has associated data in a data repository. [Authors comment: All data generated or analysed during this study are included in this published article and its supplementary information file.].

## Translations

The calculation of translations of flow fields given in Sect. 3.4 proceeds in three steps. First we express the flow fields in Cartesian coordinates, then we perform the translation (which is trivial in these coordinates), and finally, we project the translated field onto the vector spherical harmonic modes. In general, this requires Taylor expansions and elementary integrations, but for $l=1$ components, some of the results can be read off directly.

We demonstrate this procedure with a simple example. Starting from the expression for $\varvec{u}^{2<}=\frac{r}{4}\varvec{\varPsi ^2}$, given in Eq. (13), we rewrite it in terms of Cartesian coordinates

73 $$\begin{aligned} \varvec{u}^{2<}(\varvec{r})=\frac{1}{4}(z\varvec{e}_x-x\varvec{e}_z). \end{aligned}$$

The flow field in the frame of reference with origin at $\varvec{r}=\varvec{r'}+a\varvec{e}_x$ is thus given by

74 $$\begin{aligned} \varvec{u}^{2<}(\varvec{r'}+a\varvec{e}_x)^<=\frac{1}{4}(z'\varvec{e}_x-(x'+a)\varvec{e}_z)\\ =\varvec{u}^{2<}(\varvec{r'})-\frac{a}{4}\varvec{u}^{1<}(\varvec{r'}),\nonumber \end{aligned}$$

where we used the Cartesian form $\varvec{u}^{1<}=\varvec{e}_z$ to get the second of the translation relations in Sect. 3.4.

## Mobility tensors

We now give the explicit form of all the mobility tensors of the encapsulated particle and the drop as functions of $a, \eta ^\pm$ and $\epsilon$. There are 5 symmetric uniaxial tensors, $\hat{\varvec{\zeta }}_w, \hat{\varvec{\mu }}_w, \hat{\varvec{\kappa }}_D$ (with $\, w=h,F$), which possess a form analogous to Eq. (54), and 4 anti-symmetric tensors characterized by $\kappa _w, \zeta _D, \mu _D$ (see Eqs. (53, 60, 65, 66)). All these functions are polynomials of $a^2$ of degrees up to 2. The parallel components of $\hat{\varvec{\zeta }}_w$ and $\hat{\varvec{\mu }}_w$ have been obtained in I. They are included here for completeness. All other components are calculated by solving the linear system of equations set up in Sects. 3.3 and 3.5 by symbolic computing using *SymPy* [40] (except the mobility $\kappa ^{||}_D$, which is obtained in Appendix C). The *SymPy* script is added as supplementary material.

### Velocity of the squirmer

1. $$\varvec{U}=\hat{\varvec{\zeta _h}}\varvec{U}_0$$
  75 $$\begin{aligned} N\zeta ^{||}_h&= 3\epsilon ^3(\lambda -1)a^2 + C_{h0} \end{aligned}$$
  76 $$\begin{aligned} N\zeta ^\perp _h&= -\frac{3}{2}\epsilon ^3(\lambda -1)a^2 +C_{h0} \end{aligned}$$
  77 $$\begin{aligned} N=\,&2\epsilon ^5(\lambda -1) +3\lambda +2, \end{aligned}$$
  with
  78 $$\begin{aligned} C_{h0}=\,3\lambda +2 -\epsilon ^3(\lambda -1)(3\epsilon ^2-5) \end{aligned}$$
2. $$\varvec{U}=\,\hat{\varvec{\zeta _F}}\varvec{F}_{ext}$$
  79 $$\begin{aligned} N\zeta ^{||}_F&=\,\frac{1}{6\pi \epsilon \eta ^-}\big (C^{||}_4a^4 +C_{F0}\big ) \end{aligned}$$
  80 $$\begin{aligned} N\zeta ^{\perp }_F&=\,\frac{1}{6\pi \epsilon \eta ^-}\big (C^{\perp }_4a^4 + C^{\perp }_2a^2 +C_{F0}\big ) \end{aligned}$$
  with
  81 $$\begin{aligned} C_{F0}=\,&\epsilon (\lambda -1)\Big (2\epsilon ^5(\lambda -1)+\frac{9}{2}\epsilon ^4-5\epsilon ^2 \nonumber \\&+\frac{3}{2}(2\lambda + 3) \Big ) +3\lambda +2 \end{aligned}$$
  82 $$\begin{aligned} C^{||}_4 =\,&-\frac{9\epsilon }{10}(\lambda -1) \end{aligned}$$
  and
  83 $$\begin{aligned} C^{\perp }_4 =\,&-C^{||}_4 \end{aligned}$$
  84 $$\begin{aligned} C^{\perp }_2=\,&\frac{3(\lambda -1)}{4}\Big (2\epsilon ^6(\lambda -1)+5\epsilon ^3 \nonumber \\&+3\epsilon (\lambda -1)\Big ) \end{aligned}$$
3. $$\varvec{U}=\,\zeta _D \varvec{a}\times \varvec{D}_{ext}$$
  85 $$\begin{aligned} N\zeta _D=\,\frac{\lambda -1}{8\pi \eta ^-}\left( \frac{3}{2}a^2 + C_D\right) \end{aligned}$$
  with
  86 $$\begin{aligned} C_D=\,2\epsilon ^5(\lambda -1) + 5\epsilon ^2 + 3(\lambda -1) \end{aligned}$$

### Velocity of the droplet

1. $$\varvec{v}_{cm}=\,\hat{\varvec{\mu }}_h\varvec{U}_0$$
  87 $$\begin{aligned} N\mu ^{||}_h = N\mu ^{\perp }_h=\,&5\lambda \epsilon ^3 \end{aligned}$$
2. $$\varvec{v}_{cm}=\,\hat{\varvec{\mu }}_F\varvec{F}_{ext}$$
  88 $$\begin{aligned} N\mu ^{||}_F=\,&\frac{1}{12\pi \eta ^+}\Big (-3a^2 +G\Big ) \end{aligned}$$
  89 $$\begin{aligned} N\mu ^{\perp }_F=\,&\frac{1}{12\pi \eta ^+}\Big (-6a^2 +G\Big ) \end{aligned}$$
  90 $$\begin{aligned} G =\,&4\epsilon ^5(\lambda -1) - 5\epsilon ^2 + 3(2\lambda +3) \end{aligned}$$
3. $$\varvec{v}_{cm}=\mu _D \varvec{a}\times \varvec{D}_{ext}$$
  91 $$\begin{aligned} N\mu _D= \frac{-5}{8\pi \eta ^+} \end{aligned}$$

### Angular velocity of the squirmer

1. $$\varvec{\omega }= \kappa _h\varvec{a}\times \varvec{U}_0$$
  92 $$\begin{aligned} N\kappa _h=\frac{15}{2}\epsilon ^3(\lambda -1) \end{aligned}$$
2. $$\varvec{\omega }= \kappa _F\varvec{a}\times \varvec{F}_{ext}$$
  93 $$\begin{aligned} N\kappa _F= -\frac{\lambda -1}{8\pi \eta ^-}(6a^2+K) \end{aligned}$$
  with
  94 $$\begin{aligned} K=2\epsilon ^5(\lambda -1) +5 \epsilon ^2 +3(\lambda -1) \end{aligned}$$
3. $$\varvec{\omega }=\hat{\varvec{\kappa }_D}\varvec{D}_{ext}$$
  95 $$\begin{aligned} \kappa ^{||}_D=\,&\frac{1}{8\pi \eta ^-}M \end{aligned}$$
  96 $$\begin{aligned} \kappa ^{\perp }_D=\,&\frac{1}{8\pi \eta ^-}\Big (\frac{15a^2}{2N}(\lambda -1)+M \Big ) \end{aligned}$$
  with
  97 $$\begin{aligned} M= (\lambda -1) + \epsilon ^{-3} \end{aligned}$$

## Parallel alignment of displacement and torque

In I, we analyzed two uniaxial configurations: a squirmer displaced from the center of the drop such that its symmetry axis coincides with the direction of the displacement and a passive particle displaced from the center of the drop such that the applied force is parallel to the displacement. Here we discuss the extension to an applied torque, parallel to the displacement. To make use of the formalism developed in I, we choose $\varvec{D}_{ext}=D_{ext}\varvec{e}_z$ and consider a displacement $\varvec{a}=a\varvec{e}_z$. We expect a rotational velocity of the particle $\varvec{\omega }=\omega \varvec{e}_z$ and possibly a linear velocity $\varvec{U}=U\varvec{e}_z$. The flow field on the surface of the particle is given by

98 $$\begin{aligned} \varvec{v}^-(\varvec{r})=\,&\varvec{U}+\varvec{\omega }\times \varvec{r}\nonumber \\ =\,&U\varvec{e}_z+r\omega \sin {\theta }\varvec{e}_{\phi } \quad \text {for} \quad \varvec{r}\in \partial V_s. \end{aligned}$$

The contribution, due to the rotation, can be expressed in terms of vector spherical harmonics, $r\omega \sin {\theta }\varvec{e}_{\phi }=-r\omega \varvec{\varPsi ^2_{10}}$, and gives rise to a corresponding component of the flow field, $\varvec{u}^2_{10}$. We thus have to extend Eqs. (10–13) of I

99 $$\begin{aligned} \varvec{u}^{1<}:= & {} \varvec{u}^{1<}_{10}= \varvec{\varPsi }^1 \end{aligned}$$

100 $$\begin{aligned} \varvec{u}^{2<}:= & {} \varvec{u}_{10}^{2<}=\frac{r}{2}\varvec{\varPsi }_{10}^2 \end{aligned}$$

101 $$\begin{aligned} \varvec{u}^{3<}:= & {} \varvec{u}^{3<}_{10}= \frac{r^2}{3} (\varvec{\varPsi }^1+\frac{1}{5} \varvec{\varPsi }^3) \end{aligned}$$

102 $$\begin{aligned} \varvec{u}^{1>}:= & {} \varvec{u}^{1>}_{10}=\frac{1}{r^3} \varvec{\varPsi }^3 \end{aligned}$$

103 $$\begin{aligned} \varvec{u}^{2>}= & {} \varvec{u}_{10}^{2>}=-\frac{1}{r^{2}} \varvec{\varPsi }^2_{10} \end{aligned}$$

104 $$\begin{aligned} \varvec{u}^{3>}:= & {} \varvec{u}^{3>}_{10}=\frac{1}{3r} (-\varvec{\varPsi }^3 +4\varvec{\varPsi }^1) . \end{aligned}$$

The general solution of Stokes equation inside the drop is thus given by

105 $$\begin{aligned} \varvec{v}^-=a_1\varvec{u}^{1<}+a_2 \varvec{u}^{2<}+a_3\varvec{u}^{3<}+b_1\varvec{u}^{1>} +b_2\varvec{u}^{2>}+b_3\varvec{u}^{3>},\nonumber \\ \end{aligned}$$

and the flow field outside of the drop by

106 $$\begin{aligned} \varvec{v}^+=c_1\varvec{u}^{1>}++c_2 \varvec{u}^{2>}+ c_3\varvec{u}^{3>}. \end{aligned}$$

Translations in the z-direction have been worked out in I for $\varvec{u}^{1<},\varvec{u}^{3<},\varvec{u}^{1>},\varvec{u}^{3>}$ and are easily extended to the 2 new components:

107 $$\begin{aligned} \varvec{u}^{2<}(\varvec{r}-a\varvec{e}_z)= & {} \varvec{u}^{2<}(\varvec{r}) \end{aligned}$$

108 $$\begin{aligned} \varvec{u}^{2>}(\varvec{r}-a\varvec{e}_z)= & {} \varvec{u}^{2>}(\varvec{r})+{{\mathcal {O}}}(l\ge 2) \end{aligned}$$

The complete solution is now substituted in the boundary condition to determine the yet unknown coefficients. On the surface of the squirmer, Eq. (98) implies

109 $$\begin{aligned} a_1+\frac{\epsilon ^2}{3}a_3+\frac{4}{3\epsilon }b_3=&\,U \end{aligned}$$

110 $$\begin{aligned} \frac{\epsilon ^2}{15}a_3+\frac{1}{\epsilon ^3}b_1-\frac{1}{3\epsilon }b_3=&\, 0. \end{aligned}$$

111 $$\begin{aligned} \frac{\epsilon }{2}a_2-\frac{b_2}{\epsilon ^2}=&-\epsilon \omega \end{aligned}$$

On the surface of the drop, we require continuity of the flow

112 $$\begin{aligned} a_1+\frac{a_3}{3}(1+\frac{3}{5} a^2)+\frac{4}{3}(b_3-c_3)&=0 \end{aligned}$$

113 $$\begin{aligned} b_1-\frac{b_3}{3}(1-\frac{3}{5} a^2)+\frac{a_3}{15}-c_1+\frac{c_3}{3}&=0 \end{aligned}$$

114 $$\begin{aligned} \frac{a_2}{2}-b_2+c_2&=0 . \end{aligned}$$

and continuity of the tractions

115 $$\begin{aligned} \frac{a_3}{10}-b_1-\frac{a^2}{5} b_3+\frac{c_1}{\lambda }&=0 \end{aligned}$$

116 $$\begin{aligned} c_3-\lambda b_3&=0 \end{aligned}$$

117 $$\begin{aligned} 3\eta ^- b_2&= 3\eta ^+ c_2. \end{aligned}$$

These are 9 equations, which together with force and torque balance determine the 9 coefficients in the general ansatz for the flow and *U* and $\omega$. The equations simplify considerably in this case: $c_3=b_3=0$ (force balance), $c_2=D_{ext}/(8\pi \eta ^+)$ is determined by the external torque and $\lambda b_2=c_2$ (Eq. 117). This leaves us with 5 equations (110), (112), (113), (114), (115) for the remaining 5 coefficients ($a_1,a_2,a_3,b_1,c_1$), one equation for *U* (Eq. 109) and one equation for $\omega$ (Eq. 111). The result is $U=0$, i.e., no translational velocity of the squirmer; furthermore, $a_1=a_3=b_1=c_1=0$ and

118 $$\begin{aligned} \omega =\frac{D_{ext}}{8\pi \epsilon ^3\eta ^-}\big ( \epsilon ^3(\lambda -1) +1\big ) = \kappa ^{||}_D D_{ext}. \end{aligned}$$

This result is used in Eq. (64).
